# Assessment of microbiome-UV interactions in a physiological *ex vivo* human skin model

**DOI:** 10.3389/fmicb.2026.1922638

**Published:** 2026-10-28

**Authors:** Patrick Matarrese, Giuseppe Percoco, Angela Patatian, Christelle Guéré, Richard Fitoussi, Katell Vié, Valérie Poulet, Chrisse Ngari, Damien Seyer

**Affiliations:** 1 Laboratoires Clarins, Pontoise, France; 2 Eurofins BIO-EC, Longjumeau, France; 3 ERRMECe laboratory, CY Cergy-Paris Université, Cergy, France

**Keywords:** DNA damage, epidermis, *ex vivo* human skin model, oxidative stress, *Perfex* system, skin microbiome, ultraviolet radiation

## Abstract

**Introduction:**

The cutaneous microbiome is increasingly recognized as a modulator of skin physiology and UV responses, yet mechanistic studies in human models remain limited by the lack of model systems that preserve physiologically relevant skin architecture and microbial communities.

**Methods:**

We used the physiological *Perfex ex vivo* human skin explant system to assess microbiome-UV interactions. Abdominal skin explants from three donors were inoculated with donor-specific cheek microbiomes or vehicle, and, after 24 h, exposed or not to a single UV irradiation. Assessments were performed 48 h after inoculation. Bacterial survival and transfer were evaluated by culture-based CFU enumeration and 16S rRNA gene sequencing. Cutaneous responses were assessed by RT-qPCR for 29 genes involved in oxidative stress, inflammation, skin hydration, barrier function, and epidermal differentiation. Cyclobutane pyrimidine dimers (CPDs), 8-hydroxy-2′-deoxyguanosine (8-OHdG), oxidized proteins, and NRF2 were quantified by immunohistochemistry.

**Results:**

*Perfex* explants supported short-term persistence of donor-derived skin microbiota for up to 48 h, although cultivable bacteria decreased by approximately 90% over this period, including upon UV irradiation. 16S profiles confirmed donor-specific microbiome transfer but also revealed residual bacterial DNA in uninoculated controls, limiting interpretation of UV effects on microbial composition. Assessing expression changes in 29 epidermal genes examined, four—GPX1, IVL, LOR, and KRT1—exhibited UV-induced expression patterns that differed according to the presence or absence of the microbiome. By contrast, microbiome inoculation markedly attenuated UV-induced CPDs, 8-OHdG, protein oxidation, and NRF2 induction.

**Discussion:**

Despite substantial limitations in microbial engraftment, viability, and temporal resolution, the *Perfex* system provides a physiologically relevant *ex vivo* platform in which a transplanted human skin microbiome could modulate UV-induced oxidative and genotoxic damage. These proof-of-concept data support further refinement of this model for mechanistic microbiome-UV studies.

## 1 Introduction

Covering the entire body, the skin serves as a critical barrier between the organism and its environment. As the outermost organ, it is constantly exposed to external stressors, notably UV radiation (Passeron et al., 2020), whose penetration into the skin is wavelength dependent, resulting in distinct biological effects (D'Orazio et al., 2013; Tang et al., 2024). UVB radiation (280–320 nm) is largely absorbed by the epidermis where it induces erythema, edema, sunburn, and immunosuppression following acute exposure. It also induces DNA damage, predominantly cyclobutane pyrimidine dimers that can ultimately contribute to carcinogenesis. In contrast, lower-energy UVA radiation (320–400 nm) penetrates into the dermis, promoting the formation of reactive oxygen species (ROS) that indirectly damage DNA and driving photoaging manifestations such as wrinkles, pigmentary alterations, and loss of skin elasticity (Han et al., 2014; Gromkowska-Kępka et al., 2021). Although the skin has evolved efficient photoprotective mechanisms – including melanin synthesis, antioxidant defenses, and DNA repair pathways (Svobodová and Vostálová, 2010; Garcia-Mouronte et al., 2024)—these systems can be overwhelmed under excessive or chronic UV-exposure.

Yet the skin’s capacity to cope with UV stress is not solely determined by its intrinsic biology. The cutaneous microbiome—a diverse community of bacteria, fungi, viruses, and microeukaryotes residing on the epidermal surface—has emerged as a crucial regulator of skin homeostasis. Far from being a passive inhabitant, the microbiome engages in continuous crosstalk with its host (Chen et al., 2018; Cha et al., 2025), supporting fundamental physiological functions such as maintenance of barrier integrity (Ngari et al., 2025), regulation of innate and adaptive immune responses, and protection against pathogenic invasion. Its influence extends beyond the skin itself, contributing to systemic health (Zhang et al., 2024).

Given its superficial location, the skin microbiome is directly exposed to UV radiation. UV exposure has been shown to alter microbiome composition through direct damage to microbial cells and indirect effects arising from its intricate bidirectional interactions with the skin (Grant et al., 2024). Specifically, UV exposure can trigger the release of pathogen- and damage-associated molecular patterns, driving inflammation and cytokine production that modify the local microenvironment and may reshape microbiome composition by favoring certain microorganisms while suppressing others (Patra et al., 2016). This dynamic interplay between UV-induced signals, host inflammation, and immune modulation is increasingly recognized as biologically consequential, as illustrated by the demonstrated involvement of the microbiome in cutaneous UV defense and in modulating skin cancer susceptibility, progression, and therapeutic responses (Patra et al., 2020; L'Orphelin et al., 2025).

However, dissecting the mechanisms by which the microbiome modulates cutaneous responses to UV irradiation remains at an early stage, and a critical prerequisite for such studies is the availability of physiologically relevant human models that faithfully recapitulate both the skin and its associated microbiota (Boxberger et al., 2021). Two main experimental approaches have been explored toward this goal. Reconstructed human epidermis (RHE) models are well-standardized and amenable to controlled microbial inoculation (Pedrosa et al., 2019; Meloni et al., 2021; Larson et al., 2021; Cadau et al., 2022). However, they are simplified structures lacking the complexity of intact human skin, and have proven poorly conducive to long-term microbial viability (Hofmann et al., 2023; Larson et al., 2021). In contrast, *ex vivo* human skin explants preserve native skin architecture and resident immune cell populations, making them the closest available surrogate of *in vivo* human skin. They also maintain the capacity to interact with the external environment and have been used to analyze the cutaneous microbiota (Percoco et al., 2013; Mayslich et al., 2022; de Paula et al., 2024). Nevertheless, both conventional reconstructed skin models and *ex vivo* skin explants are typically maintained at 37 °C under water-saturated conditions and in a 5% CO_2_ atmosphere, conditions that alter skin biology and differ substantially from the physicochemical environment of the skin surface (Spencer et al., 1975; Borowiec et al., 2013; Danso et al., 2015; Viano et al., 2017).

To circumvent the non-physiological culture conditions described above, Peno-Mazzarino et al. (2025) developed the *Perfex* system, a human skin explant model that reproduces the physiological 37–32 °C temperature gradient from the dermis to the skin surface and operates under ambient CO_2_ (0.04%) and low relative humidity (40–60%), thereby closely mimicking the *in vivo* physicochemical environment of the skin surface.

Using this model, we recently demonstrated its capacity to support the transfer and preservation of the taxonomic diversity and donor specificity of complex aerobic and anaerobic skin bacterial communities for 48 h (Patatian et al., 2026). Building upon this skin-microbiome platform, the present study aims to investigate how the microbiome modulates the skin response to UV-irradiation. Specifically, we assessed the effects of UV-exposure on the viability and taxonomic diversity of a complex microbiome and focused primarily on how the presence of this microbiome influences the cutaneous response to UV-irradiation, assessing changes in the expression of various genes and proteins involved in barrier function, epidermal differentiation, and oxidative stress response, as well as DNA damages.

Using this model, we recently demonstrated its capacity to support the transfer and preservation of the taxonomic diversity and donor specificity of complex aerobic and anaerobic skin bacterial communities for 48 h (Patatian et al., 2026). Building upon this skin–microbiome platform, the present study aimed to investigate whether such a system could be leveraged to assess the interplay between a complex skin microbiome and UV irradiation. Specifically, we evaluated the effects of UV exposure on a donor-derived microbiome inoculated onto *Perfex* skin explants and explored whether the presence of this microbiome modulates the cutaneous response to UV irradiation, as reflected by changes in gene and protein expression and DNA damage markers. Rather than assuming full experimental maturity, the study was designed to test the feasibility of this approach, identify promising biological signals, and delineate the methodological limitations that remain to be addressed before the platform can be used for mechanistic investigation.

## 2 Materials and methods

### 2.1 Microbiome sampling

The skin microbiome was sampled from healthy female volunteers (aged 29, 35, 45, and 57 years; Fitzpatrick phototypes II-III). For each subject, samples were collected from four cheek areas of 16 cm^2^ each (two areas per cheek) using an ESwab® 480C (Copan), as previously described (Patatian et al., 2026). The samples from one individual were pooled in 1 mL of the supplied bacterial maintenance buffer.

All sampling procedures were non-invasive and conducted after obtaining written informed consent from each volunteer, in accordance with applicable national and institutional guidelines. Participation was entirely voluntary and participants were not involved in any further study procedures or follow-up.

### 2.2 Skin explants and culture conditions

Full-thickness human skin fragments were obtained from female donors (aged 25, 35, 46, and 49 years, Fitzpatrick phototype II-III) undergoing elective abdominoplasty. Tissue procurement complied with Articles L.1245–2 and L.1211–1 of the Code de la Santé Publique and written informed consent was obtained from all donors.

Following removal of the hypodermis, 38-mm-diameter skin explants were prepared. The epidermal surface was sterilized using UVC irradiation, and explants were placed in culture wells containing BIO-EC’s Explant Medium (BEM, Eurofins BIO-EC) supplemented with antibiotics. After 24 h of incubation at 37 °C under 5% CO_2_ and 90% relative humidity, the culture medium was replaced with antibiotic-free BEM, and explants were incubated for a further 48 h.

After these preliminary steps, explants were transferred to the *Perfex* reservoir, as previously described (Peno-Mazzarino et al., 2025), and maintained for the 48 h-duration of the experiment under a continuous flow of filter-sterilized air (32 °C, 0.04% CO_2_ and 40–60% relative humidity) to prevent atmospheric contamination.

### 2.3 Inoculation and UV-irradiation of skin explants

On day 0, after equilibration in the *Perfex* system, skin explants were inoculated with 150 μL of microbiome suspension deposited and spread on 452 mm^2^ of the explant, with each series of skin explants from a single donor being inoculated with the microbiome of a single subject. Uninoculated control explants received 150 μL of bacterial maintenance buffer. On day 1, half of the inoculated and uninoculated explants were exposed to UV-irradiation (UVA: 18 J/cm^2^, four times the minimum erythema dose, delivered over 3 h and 30 min; UVB: 0.3 J/cm^2^; twice the minimum erythema dose, delivered over 3 min; RMX UV simulator, Vilbert Lourmat). The remaining explants were kept in the dark for the duration of UV-exposure.

On day 2, the experiment was terminated. The cutaneous microbiome was recovered using a sterile swab soaked in bacterial maintenance buffer, following the same procedure as for initial microbiome sampling. Skin explants were divided, with one fragment preserved in RNAprotect® (Qiagen) for molecular analyses and the remaining fragment fixed in buffered formaldehyde for histological and immunohistochemical analyses.

All experimental conditions – uninoculated/unirradiated, uninoculated/irradiated, inoculated/unirradiated, and inoculated/irradiated explants – were analyzed using three technical replicates (three *Perfex* skin explants derived from the same donor and inoculated with the microbiome from the same subject). Biological replication was achieved by repeating this experimental design using skin explants from three independent donors, each inoculated with microbiome samples from three different subjects.

### 2.4 Bacterial analysis

Skin microbiome sampled from volunteers at baseline and those collected from skin explants at the end of the experiment were numerated. Aliquots of tenfold serial dilutions were spread on (i) Tryptic Soy Agar plates that were incubated aerobically at 37 °C for 48 h, and (ii) on Columbia Blood Agar plates that were incubated under anaerobic conditions (BD GasPak™ EZ, Becton Dickinson) at 37 °C for 72 h. After incubation, plates were numerated and bacterial loads were calculated considering the dilution factor and the volume plated.

Microbiomes harvested from the skin explants were also analyzed by 16S rRNA gene sequencing, using the same workflow as previously described by Patatian et al. (2026). The DNA from microbiome suspensions was extracted using a kit optimized for low mass samples (P-209B SpinColumns, DNA Genotek Inc.). The V1-V3 hypervariable region of the bacterial 16S rRNA gene was amplified and libraries were generated and sequenced on the AVITI platform (Element Biosciences, 150 bp paired-end reads). Following quality control of base quality scores and GC content, overrepresented k-mers were identified. Taxonomic classification was performed using Kraken2 against the RefSeq database. Genus-level analyses were conducted on filtered datasets (≥3 counts in ≥5% of samples) and were based on operational taxonomic unit (OTU) clustering using the phyloseq package in R.

### 2.5 Gene expression analysis

Skin explant fragments preserved in RNAprotect® were homogenized (Precellys Homogeniser) in the presence of RLT lysis buffer. After clarification by centrifugation, RNA was extracted using the RNeasy Micro Kit (Qiagen), quantified, and reverse transcribed into cDNA using RT2 First Strand Kit (Qiagen). Real-time quantitative PCR (RT-qPCR) was performed using the RT2 SYBRz Green qPCR Master Mixes (Qiagen) on a LightCyclerz 480 system (Roche) with three technical replicates per condition and biological replicates.

Gene expression analyses focused on epidermal marker genes, with glyceraldehyde-3-phosphate dehydrogenase (GAPDH), ribosomal protein L13 (RPL13), and ribosomal protein lateral stalk subunit P0 (RPLP0) used as reference genes for normalization. Relative gene expression levels were calculated using the 2^−ΔΔCt^ method, after identifying and excluding outlying Ct values using the Tukey fences (*α* < 0.05). Genes were considered differentially expressed for fold changes <0.75 or >1.5.

### 2.6 Histological and immunohistological analyses

Formaldehyde-fixed skin fragments were dehydrated through graded ethanol solutions, embedded in paraffin, and sectioned into five-micrometer-thick cross-sections.

A subset of sections was stained using Masson’s trichrome (Goldner variant) to assess possible histological alterations associated with microbiome colonization and/or UV-irradiation.

The remaining sections processed for immunohistochemical analyses. Cyclobutane pyrimidine dimers (CPDs) were detected using a mouse monoclonal antibody (Kamiya Biomedical Company, Cat# MC-062). 8-hydroxyguanosines (8-OHdGs) were labeled using a mouse monoclonal antibody (Santa Cruz Biotechnology, Cat# SC-66036,832,272). Nuclear factor erythroid 2-related factor 2 (NRF2), in its activated form (phosphor S40), was detected using a rabbit monoclonal antibody (Abcam, Cat# AB76026). These reactions were amplified and detected using the Vector VIP Substrate Kit (Vector Laboratories, Cat# SK-4600). Oxidized proteins were labeled using the OxyIHC™ Oxidative Stress Detection Kit (Sigma-Aldrich, Cat# S7450).

Stained sections were examined using a Leica DMLB, an Olympus BX63 or a Nikon Eclipse 50 microscope. Quantitative analysis of the labeled epidermal surface area was performed using the cellSens (Evident Scientific) or IPSDK Explorer (Reactiv’IP) imaging software, analyzing 9 to 20 fields per condition.

### 2.7 Statistical analysis

For every experiment, three biological donors and three explants per donor were assessed (nine explants per condition). In the present feasibility study, statistical analyses were performed at the explant level (*n* = 9 per condition). Alternative approaches, such as averaging technical replicates to obtain donor-level values (*n* = 3) or applying a mixed-effects model with donor as a random effect, were considered but not adopted: with only three donors, both approaches would leave too few degrees of freedom for reliable hypothesis testing, and a mixed-effects model would additionally require more donors to obtain a stable estimate of between-donor variance.

Results are presented as mean ± standard deviation (SD). Data normality was assessed using the Shapiro–Wilk test (*α* < 0.05). Normally distributed data were analyzed using paired t-tests or repeated-measure ANOVA, followed by Tukey HSD pairwise comparisons. Non-normally distributed data were analyzed using Wilcoxon signed rank tests or Kruskal-Wallis tests followed by *post hoc* Dunn’s tests. In the case of Tukey HSD and Dunn’s tests, *p*-values were adjusted using the Bonferroni correction, with statistical significance set at *p* < 0.05.

## 3 Results

### 3.1 Microbiome implantation and impact of UV-irradiation

To assess colonization of skin explants by donor-derived microbiomes and evaluate the effects of UV irradiation, bacterial load was quantified using culture-based colony enumeration (Table 1). Forty-eight hours after inoculation, the number of colony-forming units (CFUs) recovered from non-irradiated explants had dropped by 90.0% compared to the initial inoculum (Bonferroni-corrected *p* < 0.001), with decreases of similar magnitude for both aerobic and anaerobic bacteria. This decrease reached 99.0% of the total bacteria inoculated in UV-irradiated explants (Bonferroni-corrected *p* < 0.001). However, despite an approximately ten-fold difference, the numbers of CFUs recovered from non-irradiated and UV-irradiated explants—whether considering aerobic, anaerobic, or total bacteria—did not differ significantly (Bonferroni-corrected *p* = 0.137, *p* = 0.295, and *p* = 0.147, respectively). Finally, no CFUs were detected in uninoculated skin explants.

**Table 1:** Quantification of the number of colony forming units (CFU) inoculated on day 0 and recovered after 48 h in the different conditions evaluated.

Conditions	Bacteria quantification (CFU/cm^2^)		
	Aerobic bacteria	Anaerobic bacteria	Total bacteria
At inoculation			
Bacteria inoculated	5.69×10^3^ ± 8.83×10^2^	2.12×10^3^ ± 6.17×10^2^	7.81×10^3^ ± 7.16×10^2^
After 48 h of incubation			
Uninoculated, non-UV-irradiated controls	0	0	0
Uninoculated, UV-irradiated explants	0	0	0
Inoculated, non-UV-irradiated explants	512.2 ± 439.6***	264.4 ± 302.6**	779.6 ± 685.1***
Inoculated, UV-irradiated explants	60.4 ± 54.3***	19.2 ± 38.9**	79.6 ± 85.5***

Results are presented as mean ± standard deviation across explants (*n* = 9 per condition; three biological donors, three technical replicates per donor). Statistical analysis was performed using repeated-measure ANOVA, followed by Tukey HSD pairwise comparisons and results are presented as Bonferroni-corrected *p*-values with ***: *p* < 0.001 versus the bacterial load inoculated at study initiation.

To ensure that explant colonization reflected the composition of the inoculated microbiome and to evaluate the impact of the different experimental conditions, microbiome composition was analyzed by 16S rRNA gene sequencing (Figure 1). Despite prior UV sterilization and a 24-h incubation period in the presence of antibiotics, bacterial DNA was detected at the surface of uninoculated skin explants at the end of the experiment. Consequently, OTUs could still be identified in these samples. However, the microbial community composition of uninoculated explants differed from that of the microbiomes initially sampled from the donors’ cheek skin. The microbial community composition of UV-treated and untreated inoculated explants more closely resembled that of the inoculated microbiome, without revealing marked compositional differences attributable to UV exposure.

**Figure 1:**
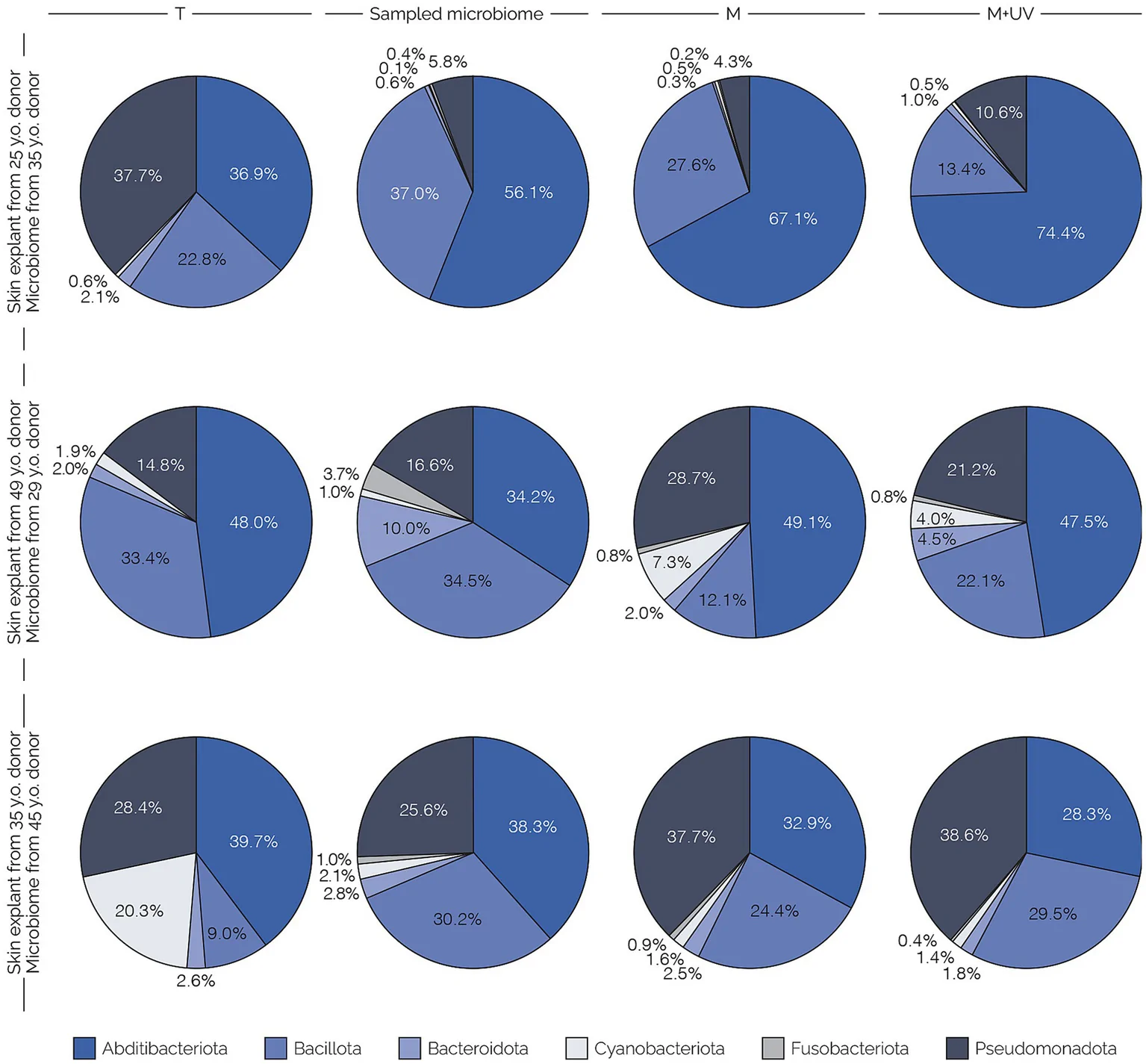
Number of different operational taxonomic units (OTUs) identified in non-UV-irradiated controls (T); microbiome sampled from the cheek of participants; inoculated, non-UV-irradiated explants (M); and inoculated, UV-irradiated explants (M + UV).

Histological evaluation using Masson’s trichrome staining revealed no detectable structural alterations in skin explants (data not shown), with no differences observed between uninoculated/unirradiated, uninoculated/irradiated, inoculated/unirradiated, and inoculated/irradiated explant.

### 3.2 Effects of the microbiome on UV-induced changes in gene expression

To analyze the effect of the microbiome on the cutaneous response to UV stress, we compared UV-induced gene expression changes in uninoculated and microbiome-inoculated explants, focusing on transcripts exhibiting fold changes <0.75 or >1.5 fold (Figure 2).

**Figure 2:**
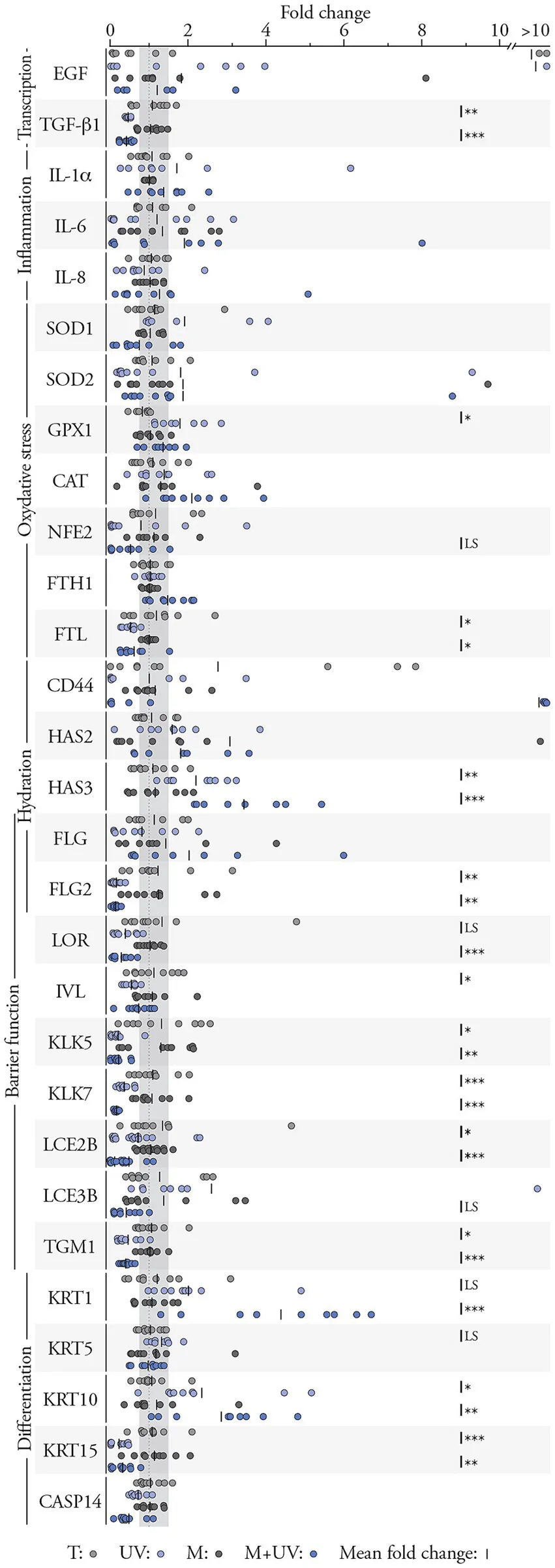
Relative expression (2^-ΔΔCt^) of genes in uninoculated, non-UV-irradiated controls (T); uninoculated, UV-irradiated explants (UV); inoculated, non-UV-irradiated explants (M); and inoculated, UV-irradiated explants (M + UV). Statistical analysis was conducted across explants (*n* = 9 per condition; three biological donors, three technical replicates per donor), using paired t-tests or Wilcoxon signed rank-test comparing T *versus* UV and M *versus* M + UV. Significance levels are only indicated for variations surpassing the <0.75- or >1.5-fold change threshold and are reported with *: *p* < 0.05, **: *p* < 0.01, and ***: *p* < 0.001.

In uninoculated explants, 24 h after UV exposure, the transcription factor TGF-β1 was downregulated, whereas EGF and the inflammation-related genes examined were not markedly affected. Most genes involved in oxidative stress responses (SOD1, SOD2, CAT, NFE2, FTH1, and FTL) showed expression levels similar to baseline, with the exception of GPX1, which was slightly upregulated. Among genes implicated in skin hydration and barrier function, FLG2, IVL, KLK5, KLK7, LCE2B, and TGM1 were downregulated. Only HAS3 was upregulated, while CD44, HAS2, FLG, LOR, and LCE3B appeared unchanged. For epidermal differentiation markers, KRT5 and CASP14 expression remained stable, KRT15 was downregulated, and KRT10 was upregulated.

Overall, microbiome-inoculated explants exhibited a broadly similar pattern of UV-induced transcriptional changes. Only four genes showed a different behavior in the presence of the microbiome: GPX1 and IVL no longer changed in response to UV, LOR became downregulated, and KRT1 was upregulated.

### 3.3 Effect of the microbiome on UV-induced changes in DNA damages markers and proteins

Changes in the cutaneous response to UV irradiation were further analyzed by quantifying DNA damage, protein oxidation, and NRF2 protein levels, comparing microbiome-inoculated and non-inoculated skin explants (Figure 3).

**Figure 3:**
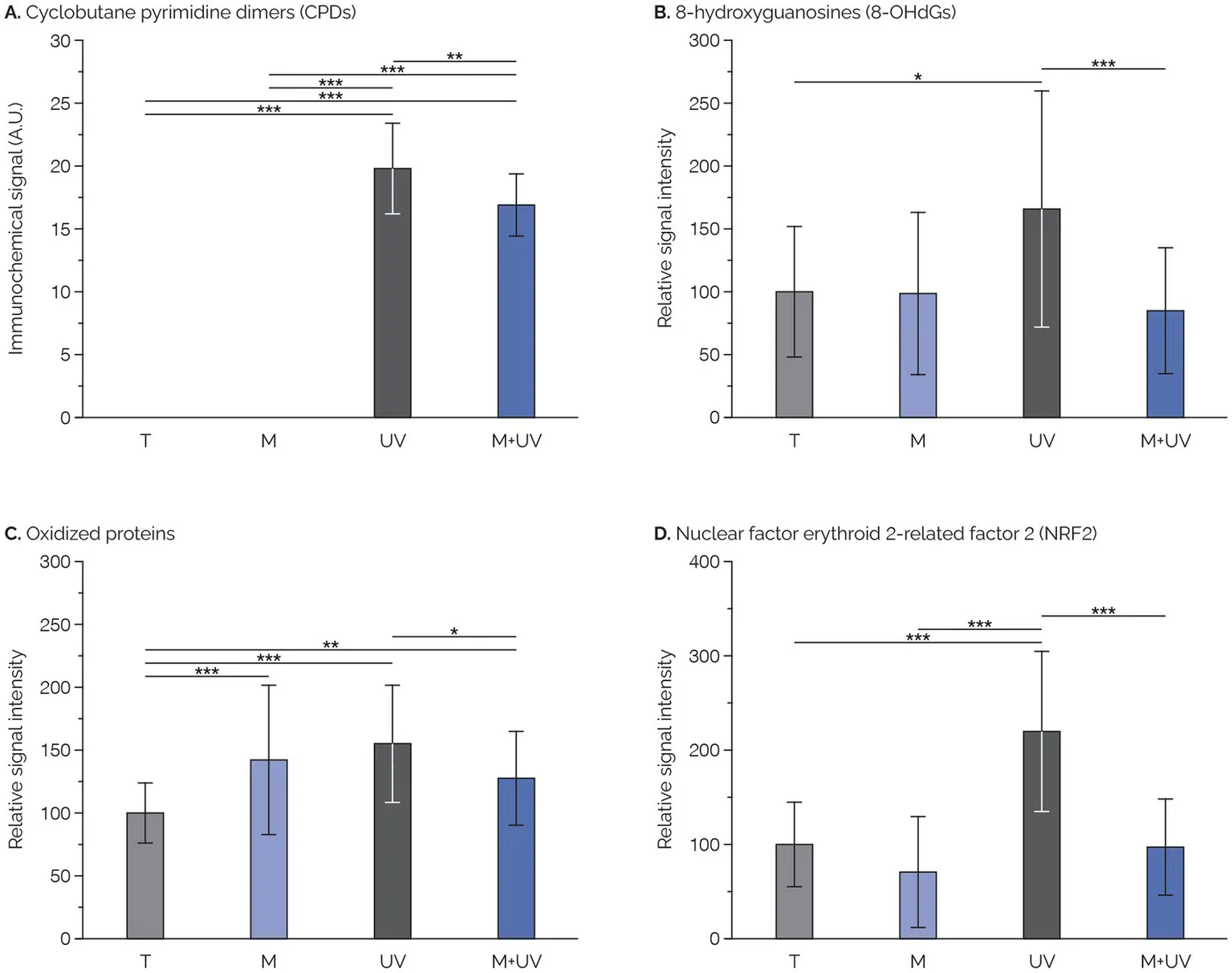
Immunohistochemical quantification of **(A)** Cyclobutane pyrimidine dimers (CPDs), **(B)** 8-hydroxyguanosines (8-OHdGs), **(C)** Oxidized proteins, and **(D)** Nuclear factor erythroid 2-related factor 2 (NRF2) in uninoculated, non-UV-irradiated controls (T); uninoculated, UV-irradiated explants (UV); inoculated, non-UV-irradiated explants (M); and inoculated, UV-irradiated explants (M + UV). Statistical analysis was conducted across explants (*n* = 9 per condition; three biological donors, three technical replicates per donor), using repeated-measure ANOVA and Tukey HSD or Tukey HSD and Dunn’s tests. Bonferroni-corrected significance levels are reported with *: *p* < 0.05, **: *p* < 0.01, and ***: *p* < 0.001.

CPDs were absent in non-UV-irradiated explants. Upon UV exposure, CPD levels were lower in microbiome-inoculated, UV irradiated explants than in non-inoculated ones subjected to UV irradiation (−14%, Bonferroni-corrected *p* = 0.010). 8-OHdG levels were similar in microbiome-inoculated explants subjected to UV irradiation and in non-irradiated skin explants, irrespective of microbiome inoculation. In contrast, 8-OHdG levels were higher in non-inoculated explants exposed to UV stress (+49% compared to inoculated explants subjected to UV stress, Bonferroni-corrected *p* < 0.001).

Compared with uninoculated, non-UV-irradiated explants, microbiome inoculation increased oxidized protein levels (+42%, Bonferroni-corrected *p* < 0.001) to values that were not further modified by subsequent UV irradiation (+4%, Bonferroni-corrected *p* = 1.0 *versus* inoculated, non-irradiated explants). UV-irradiated explants also exhibited higher levels of oxidized proteins when non-inoculated than when inoculated (+18%, Bonferroni-corrected *p* = 0.025).

NRF2 (phospho-S40, active form) levels in inoculated, non-irradiated explants and inoculated, irradiated explants were comparable to those observed in non-inoculated, non-irradiated explants. Only UV irradiation of non-inoculated explants resulted in a significant increase in NRF2 expression (+120% compared with non-inoculated, non-irradiated explants, Bonferroni-corrected *p* < 0.001).

## 4 Discussion

The present study aimed to investigate the feasibility of using the *Perfex ex vivo* skin explant model as a platform for assessing microbiome-UV interactions. Before interpreting the biological effects of the microbiome on the cutaneous response to UV irradiation, it is important to consider the conditions under which microbial colonization was achieved and the limitations of the approaches used to characterize it.

Colony-forming unit enumeration confirmed that viable bacteria were present on skin explants 48 h after inoculation, establishing that the *Perfex* model supports short-term bacterial survival. In non-irradiated skin explants, approximately 90% of cultivable bacteria were lost over the course of the 48-h incubation, a finding consistent with that reported by Patatian et al. (2026) using the same experimental design. This convergence is noteworthy given that microbiome sampling efficiency was approximately ten-fold lower in the present study, resulting in a correspondingly reduced initial inoculum. The fact that a similar relative attrition was observed despite this difference in starting bacterial load suggests that the 90% loss reflects a reproducible, intrinsic feature of the model rather than experimental variability.

Several, not mutually exclusive, mechanisms may underlie this reproducible one log decline. Physicochemical constraints intrinsic to the *Perfex* system are likely to limit sustained microbial growth and favor attrition over time. In addition, both cross-site and inter-donor aspects of the microbiome transplantation may have contributed to the poor engraftment. Cheek skin hosts a relatively low-diversity, *Cutibacterium*-dominated community adapted to a sebum-rich environment, and topographical surveys of the skin microbiome indicate that body sites with distinct biophysical characteristics, such as the cheek and the abdomen, harbor compositionally different bacterial communities (Mukherjee et al., 2016; Costello et al., 2009; Grice and Segre, 2011), suggesting that a microbiome sampled from one site may be poorly suited to colonize another. Consistent with this, experimental work on skin microbiome transplantation indicates that intra-individual cross-site transfers often result in only partial and short-lived engraftment, even under optimized conditions, with the recipient-site microenvironment exerting a strong selective filter (Perin et al., 2019; Callewaert et al., 2021). Furthermore, inter-individual transplantation, even at the same anatomical site, has yielded incomplete and sometimes transient engraftment (Callewaert et al., 2021), and twin-based analyses indicate that host genetics contribute to skin microbiome composition (Si et al., 2015), suggesting that donor-recipient compatibility and host-specific factors are likely to further influence colonization success. Future studies using anatomically matched donor and recipient sites—sampling the abdominal skin microbiome directly rather than transplanting a cheek-derived microbiome onto abdominal explants—may help disentangle site-mismatch effects from other sources of engraftment failure.

Regardless of the underlying mechanisms, the practical consequence of the bacterial loss is that, at the end of the 48-h incubation, only a limited number of viable bacteria remained on the surface of non-UV-irradiated explants. Whether the approximately ten-fold reduction observed in UV-irradiated explants reflects a genuine bactericidal effect of UV irradiation or simply experimental variability cannot be determined at this stage, because the very low bacterial counts leave the assay underpowered. More broadly, the absence of time-course data raises a fundamental question about the experimental design itself: bacterial load at the time of UV irradiation—24 h after inoculation—is unknown, and it is therefore unclear how many viable bacteria were present on the skin surface when the UV response was initiated. This is particularly relevant given that microbiome–skin crosstalk involves a range of soluble mediators and metabolites produced through direct interactions (Chen et al., 2018; Chinnappan and Harris-Tryon, 2021), and quorum-sensing systems in skin-associated *Staphylococci* provide a well-characterized example of density-dependent regulation of microbial activities at the skin surface (Williams et al., 2019; Williams et al., 2023). Therefore, the bacterial load at the time of UV exposure is likely to determine the amplitude of any modulatory effect to the UV stress. Establishing detailed bacterial survival kinetics on the *Perfex* system across multiple time points will be essential to build solid foundations that will enhances the platform’s power for robust mechanistic exploration of microbiome and UV interactions. At this stage, it remains impossible to determine whether the protective effects observed in microbiome-inoculated explants arise from viable bacteria, residual microbial biomass, bacterial metabolites, or a combination of these factors.

In the present study, 16S rRNA gene sequencing and OTU analysis revealed that the community detected on inoculated explants more closely resembled the donor cheek microbiome than the signal detected on uninoculated controls, supporting the successful transfer of the donor-derived microbiome onto the explants. This finding is consistent with the more extensive characterization reported by Patatian et al. (2026), who demonstrated that microbiome transplantation to *Perfex* explants results in successful engraftment with preservation of donor-specific taxonomic signatures. However, OTUs were also detected in uninoculated, non-irradiated control explants despite pre-culture in antibiotic-containing medium and UV sterilization of the epidermal surface, although no cultivable bacteria could be recovered from these controls at the end of the experiment. This indicates that the 16S workflow captures residual bacterial DNA from the original explant microbiome long after bacteria have died. Under such conditions, any subtle UV-induced change in the OTU composition of the living bacterial fraction is effectively masked by remaining DNA from bacteria that may have been killed during the UV exposure or earlier in the incubation. Besides, our analysis relied on OTU clustering rather than amplicon sequence variants (ASVs); given the very low bacterial biomass remaining at 48 h. However, pushing resolution to the strain level would likely have amplified noise rather than yielded additional biological insight, and downstream analyses were therefore conservatively restricted to the genus level. Overcoming the residual-DNA limitation would require restricting sequencing to viable cells—for example, by coupling viability-based cell sorting to DNA extraction—and, critically, achieving substantially higher bacterial loads at 48 h to ensure that such an approach, along with ASV-based or higher-resolution methods, is analytically robust.

It is important to note that, as outlined above, the bacterial load actually present on the skin surface at the time of UV exposure (24 h after inoculation) was not determined. This uncertainty applies equally to the gene expression and immunohistochemical damage marker results discussed below, and the mechanistic interpretations of both sets of findings should be considered in light of this unquantified bacterial dose.

UV irradiation is known to alter the expression of multiple genes in human skin (Enk et al., 2006), with consistent effects reported for transcription factors, pro-inflammatory cytokines, and antioxidant defense enzymes (Quan et al., 2002; Chung et al., 1996; Strickland et al., 1997; Marionnet et al., 2010). Beyond these canonical stress and inflammatory pathways, UV exposure also affects genes involved in epidermal functions such as hyaluronic acid metabolism, barrier and cornified envelope formation, kallikrein-type serine proteases, late cornified envelope proteins, and epidermal differentiation (Nin et al., 2009; Bernerd and Asselineau, 1997; Huang et al., 2017; Simonsen et al., 2017). In our study, analysis of 29 genes spanning these pathways showed that 15 showed no significant UV-induced differential expression, and 10 were differentially expressed upon UV irradiation but showed an identical pattern regardless of microbiome inoculation. Only four genes—GPX1, IVL, LOR, and KRT1—showed a UV-induced expression profile that differed according to microbiome status, indicating at most a modest microbiome-dependent effect under the present experimental conditions.

Focusing specifically on the four genes whose UV-induced expression differed according to the presence of the microbiome, GPX1 was modestly overexpressed 24 h after a single UV exposure. Although biologically plausible as an antioxidant response, this contrasts with *in vivo* human data showing reduced GPX1 expression after UV, albeit under a chronic exposure (Enk et al., 2006). IVL was slightly downregulated, consistent with reports of transient suppression in UVB-irradiated HaCaT keratinocytes (Moravcová et al., 2013), whereas no significant change was observed in reconstructed human epidermis under comparable conditions (Bernerd and Asselineau, 1997). LOR and KRT1 expression was not significantly affected, which is compatible with their roles in the late stages of differentiation and in structurally stable cornified envelopes. Although KRT1 upregulation has been reported in UVB-irradiated HaCaT keratinocytes (Moravcová et al., 2013), to the best of our knowledge no RHE, *ex vivo* skin explant, or *in vivo* study has reported LOR or KRT1 gene expression changes 24 h after a single, physiologically relevant UV exposure.

In microbiome-inoculated explants, UV-induced GPX1 and IVL changes were no longer observed, suggesting that the resident microbiome may modulate the keratinocyte antioxidant and differentiation response to UV. Conversely, UV exposure in inoculated explants led to LOR downregulation and KRT1 upregulation, suggesting a microbiome-dependent shift in the epidermal differentiation program, with impaired cornified envelope maturation and enhanced structural keratin expression.

Nevertheless, these results should be interpreted with extreme caution as several methodological limitations are likely to have constrained the sensitivity of this analysis. Gene expression analysis focused on predominantly epidermal transcripts, yet used RNA extracted from full-thickness skin explants. These explants contain multiple cell types. Although keratinocytes are abundant (Hybbinette et al., 1999; Guo et al., 2024), the transcripts of epidermal genes constitute only a fraction of the total RNA pool (Bustamante et al., 2020), a known source of increased variability in RT-qPCR results (Ståhlberg et al., 2004). Besides, three independent skin donor-microbiome donor pairs were used. While this design was interesting to capture biological variability, it likely introduced additional sources of variability arising from inter-individual differences in baseline gene expression, inter-donor microbiome composition diversity, and engraftment efficiency, all of which would require a substantially larger experimental design to disentangle. In this context, the targeted 29-gene panel, while covering key epidermal functions known to be UV-responsive, represents only a small fraction of the transcriptional landscape that UV irradiation and microbiome-skin crosstalk are likely to engage. Moreover, the use of only three biological replicates further limits the statistical power to distinguish true microbiome-dependent changes in gene expression from random variation. Additionally, the four genes identified as differentially responsive to UV depending on microbiome status were selected by comparing UV-induced changes separately within uninoculated and inoculated explants, rather than through a single comparison with correction for multiple testing. They should therefore be regarded as candidate markers warranting validation in future studies, rather than definitive findings. Finally, gene expression was assessed at a single time point (24 h post-irradiation), whereas the UV-induced transcriptional response is temporally structured (Bustamante et al., 2020; Enk et al., 2006). Our approach therefore risks missing both early microbiome-dependent regulation and later response phases. A time-course design sampling several time points—for example 4, 24, and 48 h post-irradiation—would be more informative, but is currently precluded by the limited availability of sufficiently large surgical skin discards and of cheek-derived microbiome material from a single donor. More broadly, across all analyses presented in this study, statistical analyses were conducted at the explant level (*n* = 9), treating technical replicates from the same donor as independent observations, a form of pseudoreplication that may have inflated significance.

Strikingly, all markers assessed by immunohistological analysis—CPDs, 8-OHdG, oxidized protein, and nuclear phospho-S40 NRF2 protein—are all appearing within one or a few hours after a single exposure and remain present for at least 24 h (Ahmed et al., 1999; Ryšavá et al., 2020; Fajuyigbe et al., 2021; Cho et al., 2022). Thus, the changes observed at the end of the 48-h incubation reflect early UV-induced events captured due to their persistence. Strikingly, these data show that the presence of the skin microbiome substantially attenuated UV-induced oxidative and genotoxic damage and blunted the associated NRF2 response, in line with the concept that reducing the oxidative burden diminishes the need for stress-induced NRF2 activation. Notably, microbiome inoculation alone raised baseline oxidized protein levels, a pattern consistent with oxidative preconditioning, whereby a mild, tolerable oxidative challenge from bacterial colonization primes host antioxidant defenses and confers protection against a subsequent, larger insult such as UV exposure (Andersson et al., 2019). Alternative, non-exclusive explanations include oxidative by-products of bacterial metabolism and stress responses associated with colonization and the inoculation procedure. From a mechanistic standpoint, both biochemical effects (microbiome-derived metabolites or colonization-induced stress modulating redox homeostasis or DNA repair) and a purely physical effect (microbial biomass acting as a partial UV filter) also remain plausible and cannot be distinguished based on the present data. Indeed, the reduction observed across all four damage markers—CPDs, 8-OHdG, oxidized proteins, and NRF2 activation—is equally consistent with a simple decrease in UV penetration into the tissue afforded by the bacterial biomass deposited on the explant surface. Distinguishing a biologically active protective mechanism from passive physical shielding would require dedicated control conditions not included in the present study, such as explants inoculated with UV-killed microbiota. Incorporating such controls should be a priority in future iterations of this experimental design. Nevertheless, the attenuation of CPDs, 8-OHdG, protein oxidation, and NRF2 induction in microbiome-inoculated explants indicates that the bacterial load present at the time of UV exposure—24 h after inoculation—was sufficient to measurably modulate the cutaneous response, thereby supporting the feasibility of using the *Perfex* system to probe microbiome-dependent effects on UV-induced damage under well-controlled conditions.

Taken together, these findings indicate that, in this early exploratory phase, the *Perfex ex vivo* skin explant system provides a physiologically relevant platform for probing microbiome-UV interactions in human skin. Technical constraints, such as suboptimal engraftment, low final bacterial loads, and the lack of time-resolved microbiome quantification, currently preclude a comprehensive analysis of UV effects on the microbiome itself and of microbiome-dependent transcriptional reprogramming in the skin. Nevertheless, the attenuation of UV-induced CPDs, 8-OHdG, protein oxidation, and NRF2 induction in microbiome-inoculated explants would indicate that, under the present conditions, the transplanted microbiome could modulate the cutaneous response to UV. Future refinements aimed at improving bacterial survival, increasing inoculum size, and implementing longitudinal designs with viability-focused microbiome profiling should further enhance the utility of this model for mechanistic studies of microbiome-mediated photoprotection.

## Data Availability

The datasets presented in this study can be found in online repositories. The names of the repository/repositories and accession number(s) can be found at: https://www.ebi.ac.uk/metagenomics/, PRJEB121510.
